# Diaqua­bis[1-hydroxy-2-(imidazol-3-ium-1-yl)-1,1′-ethyl­idenediphophonato-κ^2^ *O*,*O*′]zinc(II)

**DOI:** 10.1107/S1600536809042858

**Published:** 2009-10-23

**Authors:** Eleonora Freire, Daniel R. Vega

**Affiliations:** a Gerencia de Investigación y Aplicaciones, Centro Atómico Constituyentes, Comisión Nacional de Energía Atómica and, Escuela de Ciencia y Tecnología, Universidad Nacional General San Martín, Buenos Aires, Argentina

## Abstract

In the title complex, [Zn(C_5_H_9_NO_7_P_2_)_2_(H_2_O)_2_], the zinc atom is coordinated by two bidentate zoledronate [zoledronate = (2-(1-imidazole)-1-hydr­oxy-1,1′-ethyl­idenediphophonate)] ligands and two water mol­ecules. The coordination number is 6. There is one half-mol­ecule in the asymmetric unit with the zinc atom located on a crystallographic inversion centre. The anion exists as a zwitterion with an overall charge of −1; the protonated nitro­gen in the ring has a positive charge and the two phospho­nates groups each have a single negative charge. There are two intra­molecular O—H⋯O hydrogen bonds. The mol­ecules are linked into a chain by inter­molecular O—H⋯O hydrogen bonds. Adjacent chains are further linked by O—H⋯O hydrogen bonds involving the aqua ligands. An N—H⋯O inter­action is also observed.

## Related literature

For general background to bis­phospho­nates, see: Fleisch *et al.* (1968 ▶); Green *et al.* (1994 ▶); Fleisch (2000 ▶); Ross *et al.* (2004 ▶); Smith (2005 ▶); Ralston *et al.* (1989 ▶); Reid *et al.* (2005 ▶); Rauch & Glorieux (2005 ▶); Chesnut *et al.* (2004 ▶). For structures of transition metal (Ni, Co and Cu) complexes with the zoledronate anion, see: Cao *et al. *(2007*, *2008). For metal complexes of other bis­phospho­nates (Etidronate and Pamidronate), see: Fernández *et al.* (2002 ▶); Li *et al.* (2008 ▶); Chen *et al.* (2008 ▶); Uchtman (1972 ▶). For a penta­coordinated zinc(II)–zoledronate complex, see: Freire & Vega (2009 ▶). For bond distances and angles in related structures, see: Coiro & Lamba (1989 ▶); Vega *et al.* (1996 ▶, 1998 ▶). For hydrogen-bond motifs, see: Bernstein *et al.* (1995 ▶).
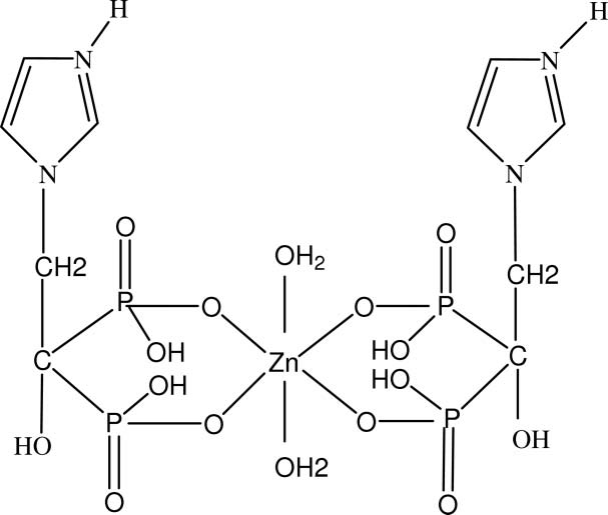


## Experimental

### 

#### Crystal data

[Zn(C_5_H_9_N_2_O_7_P_2_)_2_(H_2_O)_2_]
*M* *_r_* = 643.57
Triclinic, 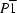

*a* = 7.457 (1) Å
*b* = 8.408 (2) Å
*c* = 9.843 (2) Å
α = 105.06 (3)°
β = 112.23 (3)°
γ = 97.05 (3)°
*V* = 534.5 (2) Å^3^
*Z* = 1
Mo *K*α radiation
μ = 1.54 mm^−1^
*T* = 293 K
0.18 × 0.11 × 0.05 mm

#### Data collection

Rigaku AFC6 diffractometer diffractometer
Absorption correction: ψ scan (North *et al.*, 1968 ▶) *T*_min_ = 0.82, *T*_max_ = 0.92
2426 measured reflections
1990 independent reflections
1236 reflections with *I* > 2σ(*I*)
*R*_int_ = 0.050
3 standard reflections every 150 reflections intensity decay: <3%

#### Refinement

*R*[*F* ^2^ > 2σ(*F* ^2^)] = 0.052
*wR*(*F* ^2^) = 0.152
*S* = 1.04
1990 reflections
160 parameters
H-atom parameters constrained
Δρ_max_ = 0.84 e Å^−3^
Δρ_min_ = −0.94 e Å^−3^

### 

Data collection: *MSC/AFC Diffractometer Control Software* (Molecular Structure Corporation, 1988 ▶); cell refinement: *MSC/AFC Diffractometer Control Software*; data reduction: *MSC/AFC Diffractometer Control Software*; program(s) used to solve structure: *SHELXS97* (Sheldrick, 2008 ▶); program(s) used to refine structure: *SHELXL97* (Sheldrick, 2008 ▶); molecular graphics: *SHELXTL* (Sheldrick, 2008 ▶); software used to prepare material for publication: *SHELXTL* and *PLATON* (Spek, 2009 ▶).

## Supplementary Material

Crystal structure: contains datablocks global, I. DOI: 10.1107/S1600536809042858/bq2165sup1.cif

Structure factors: contains datablocks I. DOI: 10.1107/S1600536809042858/bq2165Isup2.hkl

Additional supplementary materials:  crystallographic information; 3D view; checkCIF report

## Figures and Tables

**Table:** 

Zn1—O11	2.042 (4)
Zn1—O21	2.079 (4)
Zn1—O1*W*	2.096 (4)

**Table:** 

O11—Zn1—O21	90.65 (16)
O11—Zn1—O1*W*	86.18 (18)
O21—Zn1—O1*W*	92.63 (18)

**Table 2:** Hydrogen-bond geometry (Å, °)

*D*—H⋯*A*	*D*—H	H⋯*A*	*D*⋯*A*	*D*—H⋯*A*
O22—H22⋯O23^i^	0.82	1.90	2.676 (6)	159
O12—H12⋯O13^ii^	0.82	1.79	2.607 (6)	176
O1—H1⋯O23^i^	0.82	2.28	2.910 (6)	134
O1*W*—H1*WA*⋯O12	0.82	2.43	3.078 (6)	137
O1*W*—H1*WB*⋯O13^iii^	0.82	1.94	2.745 (6)	167
N2—H2⋯O21^iv^	0.86	1.90	2.740 (7)	164

Symmetry codes: (i) 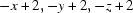; (ii) 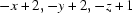; (iii) 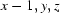; (iv) 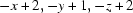.

**Fig. 1.**
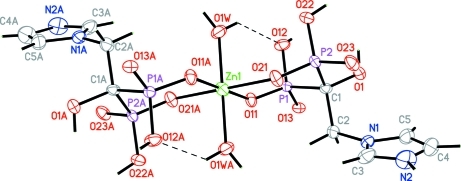
: Molecular view of (I), showing the labeling scheme used. Hydrogen bonding is shown in dashed lines.

**Fig. 2.**
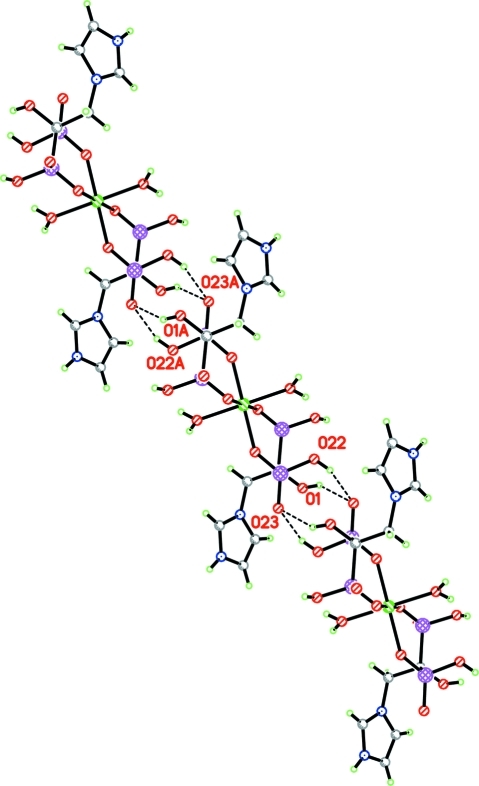
: View of the intra chain H-bonds in (I).

## supplementary crystallographic information

### Comment

The present work is part of a project directed to the preparation and characterization of coordination complexes obtained by the interaction among metals and organic molecules of relevant pharmacological interest like bisphosphonates. Bisphosphonate compounds, which are characterized by a P—C—P backbone, are analogues of naturally occurring pyrophosphates. As effective inhibitors of bone resoption, bisphosphonates are used in the treatment of various bone diseases and disorders of calcium metabolism, osteolityc tumor bone disease, non tumor induced hypercalcemia, Paget and osteoporosis (Fleisch, 2000; Ross *et al.*, 2004; Smith, 2005; Ralston *et al.*, 1989; Reid *et al.*, 2005; Rauch *et al.*, 2005 and Chesnut *et al.*, 2004). The P—C—P base structure allows the bisphosphonates to bind to many metallic cations in particular divalent metal ions and as a result bisphosphonates may stick bone surfaces *in vivo* (Fleisch *et al.*, 1968). Third generation bisphosphonates, like zoledronate, are characterized by having a cyclic side chain and belong to the nitrogen containing bisphosphonate group which are the most effective for medical treatment (Green *et al.*, 1994). A large number of metal derivatives of other bisphosphonates, like Etidronate and Pamidronate are known where the bisphosphonate ligand displays a variety of coordination modes (Ferńandez, 2002; Li *et al.*, 2008; Chen *et al.*, 2008; Uchtman, 1972). In contrast, few metal derivatives of Zoledronic acid have been reported in CSD (Allen, 2002). The present compound is isostructural with two Co and Ni complexes (Cao *et al.*, 2007).

So, we present herein the crystal structure of a Zinc-Zoledronate complex: monozinc dizoledronate dihydrate, (I), Zn.2(P_2_O_7_N_2_C_5_H_9_).2(H_2_O). We also synthesized a pentacoordinated complex of zinc (II), (Freire & Vega, 2009). The zoledronate anion exists as a zwitterion with an overall charge of -1; the protonated nitrogen in the ring has a positive charge and the two phosphonates groups each have a single negative charge.

The ZnO_6_ coordination sphere (Fig. 1) is defined by O11, O21, O1W and and their (1 - *x*, 1 - *y*, 1 - *z*) counterparts generated by the inversion center on Zn1. Zn – O distances range from 2.041 (4) to 2.095 (4) Å, and octahedral angles between 86.2 (2) and 92.7 (2)°. The O11—Zn1—O21 zoledronate bite angle is 90.6 (2)° and the angle between two oxygen atoms of different zoledronates is O11A Zn1 O21 89.4 (2)°.

Each phosphonate has one protonated O atom, the extra electronic charge being shared by the remaining two non protonated O atoms. This fact define two distinct types of P—O bonds, as shown by the mean value in the following values of bond distances and angles: P—OH 1,572 (8), P - O 1.503 (5) Å, O—P—OH 109.45 (13), O—P—O 115.60 (14) °, this measure is in agreement with the results found for related molecules (Coiro *et al.*, 1989; Vega *et al.*, 1996; Vega *et al.*, 1998). The phosphonates groups have slighty "staggered" conformations sight in the P1···P2 direction. When this staggering is observed the non bonded torsion angle O13—P1··· P2—O23 is 4.1 °. The imidazol ring is plane, maximum deviation from the L.S. mean plane is 0.0058 Å for C5. The ring and C2 are not coplanar, the angle determined between the plane of the ring and the bond N1—C2 is 3.2 ° and C2 is 0.0861 Å far from the plane of the ring. The torsion angle C1—C2—N1—C3 is of 104.1 (8) ° and it is possible to describe it like + Anti-Clinal (+*ac*).

In this compound there are two intramolecular hydrogen bonds O1W—H1WA···. O12 and the one generated by the center of symmetry (Fig. 1).  These molecules form a chain by means of hydrogen bonds provided by O22—H22··· O23 (-*x* + 2,-*y* + 2,-*z* + 2) and O1—H1··· O23 (-*x* + 2,-*y* + 2,-*z* + 2) (Fig. 2). This chain joins other neighboring and similar chains by means of the hydrogen bonds O1W—H1WB··· O13 (*x* - 1, *y*, *z*), O12—H12··· O13 (-*x* + 2,-*y* + 2,-*z* + 1), and N2—H2··· O21 (-*x* + 2,-*y* + 1,-*z* + 2) (see Table 2), determining a three-dimensional net. In all this hydrogen-bonding network the presence of two homodromic rings (Bernstein *et al.*, 1995) is observed: P2—O22—H22··· O23 (-*x* + 2,-*y* + 2,-*z* + 2) ···H1—O1—C1—P2 (*R*_2_^1^(7)), P1—O12—H12··· O13—P1—O12—H12 (-*x* + 2,-*y* + 2,-*z* + 1)··· O13—P1 (*R*_2_^2^(8)).

### Experimental

Zoledronic Acid was obtained from Gador S. A. laboratory. The present compound was obtained as subproduct in a Zoledronate recrystallization process.

### Refinement

The H atoms attached to O were found in a difference Fourier map, further idealized (O—H: 0.82 Å - 0.90 Å) and finally allowed to ride. Those attached to C and N were placed at calculated positions (C—H: 0.93 Å; C—H_2_: 0.97 Å; N—H_2_: 0.90 Å) and allowed to ride. Displacement factors were taken as U(H)_isot_ = *x*.U(host), *x*: 1.2 (C—H); 1.5 (C—H_2_, N—H_2_, O—H).

### Crystal data

**Table:**

[Zn(C_5_H_9_N_2_O_7_P_2_)_2_(H_2_O)_2_]	*Z* = 1
*M**_r_* = 643.57	*F*(000) = 328
Triclinic, *P*1	*D*_x_ = 1.999 Mg m^−^^3^
Hall symbol: -P 1	Mo *K*α radiation, λ = 0.71073 Å
*a* = 7.457 (1) Å	Cell parameters from 42 reflections
*b* = 8.408 (2) Å	θ = 8–18°
*c* = 9.843 (2) Å	µ = 1.54 mm^−^^1^
α = 105.06 (3)°	*T* = 293 K
β = 112.23 (3)°	Prism, colorless
γ = 97.05 (3)°	0.18 × 0.11 × 0.05 mm
*V* = 534.5 (2) Å^3^	

### Data collection

**Table:**

Rigaku AFC6 Difractometer diffractometer	1236 reflections with *I* > 2σ(*I*)
Radiation source: fine-focus sealed tube	*R*_int_ = 0.050
graphite	θ_max_ = 25.5°, θ_min_ = 2.4°
ω/2θ scans	*h* = −9→9
Absorption correction: ψ scan (North *et al.*, 1968)	*k* = −1→10
*T*_min_ = 0.82, *T*_max_ = 0.92	*l* = −11→11
2426 measured reflections	3 standard reflections every 150 reflections
1990 independent reflections	intensity decay: <3%

### Refinement

**Table:**

Refinement on *F*^2^	Primary atom site location: structure-invariant direct methods
Least-squares matrix: full	Secondary atom site location: difference Fourier map
*R*[*F*^2^ > 2σ(*F*^2^)] = 0.052	Hydrogen site location: inferred from neighbouring sites
*wR*(*F*^2^) = 0.152	H-atom parameters constrained
*S* = 1.04	*w* = 1/[σ^2^(*F*_o_^2^) + (0.0781*P*)^2^] where *P* = (*F*_o_^2^ + 2*F*_c_^2^)/3
1990 reflections	(Δ/σ)_max_ < 0.001
160 parameters	Δρ_max_ = 0.84 e Å^−^^3^
0 restraints	Δρ_min_ = −0.94 e Å^−^^3^

### Special details

**Table:**

Geometry. All e.s.d.'s (except the e.s.d. in the dihedral angle between two l.s. planes) are estimated using the full covariance matrix. The cell e.s.d.'s are taken into account individually in the estimation of e.s.d.'s in distances, angles and torsion angles; correlations between e.s.d.'s in cell parameters are only used when they are defined by crystal symmetry. An approximate (isotropic) treatment of cell e.s.d.'s is used for estimating e.s.d.'s involving l.s. planes.

### Fractional atomic coordinates and isotropic or equivalent isotropic displacement parameters (Å^2^)

**Table:**

	*x*	*y*	*z*	*U*_iso_*/*U*_eq_	
Zn1	0.5000	0.5000	0.5000	0.0266 (4)	
P1	0.8731 (2)	0.77240 (19)	0.52403 (17)	0.0172 (4)	
P2	0.8422 (2)	0.77671 (19)	0.82873 (17)	0.0186 (4)	
O11	0.7109 (6)	0.6106 (5)	0.4453 (4)	0.0211 (9)	
O12	0.7749 (6)	0.9268 (5)	0.5228 (5)	0.0242 (10)	
H12	0.8403	1.0149	0.5264	0.036*	
O13	1.0242 (6)	0.7849 (5)	0.4574 (5)	0.0217 (9)	
O21	0.6875 (6)	0.6116 (5)	0.7361 (5)	0.0253 (10)	
O22	0.7342 (6)	0.9253 (5)	0.8129 (5)	0.0254 (10)	
H22	0.8021	1.0216	0.8691	0.038*	
O23	0.9654 (7)	0.8002 (5)	0.9969 (5)	0.0249 (10)	
O1	1.1578 (6)	0.9545 (5)	0.8097 (5)	0.0261 (10)	
H1	1.1019	1.0326	0.8140	0.039*	
N1	1.2533 (8)	0.6520 (6)	0.8864 (6)	0.0224 (11)	
N2	1.3599 (9)	0.5904 (8)	1.0957 (7)	0.0372 (15)	
H2	1.3678	0.5388	1.1618	0.045*	
C1	1.0088 (9)	0.7937 (7)	0.7310 (6)	0.0183 (13)	
C2	1.1243 (10)	0.6535 (8)	0.7302 (7)	0.0271 (15)	
H2A	1.0288	0.5439	0.6719	0.032*	
H2B	1.2067	0.6680	0.6759	0.032*	
C3	1.2149 (10)	0.5438 (9)	0.9533 (8)	0.0315 (16)	
H3	1.1052	0.4509	0.9084	0.038*	
C5	1.4298 (10)	0.7711 (8)	0.9896 (8)	0.0319 (16)	
H5	1.4929	0.8611	0.9722	0.038*	
C4	1.4926 (12)	0.7303 (10)	1.1212 (8)	0.042 (2)	
H4	1.6068	0.7885	1.2127	0.051*	
O1W	0.3851 (6)	0.7142 (6)	0.4904 (6)	0.0349 (12)	
H1WA	0.4645	0.8074	0.5273	0.052*	
H1WB	0.2703	0.7245	0.4668	0.052*	

### Atomic displacement parameters (Å^2^)

**Table:**

	*U*^11^	*U*^22^	*U*^33^	*U*^12^	*U*^13^	*U*^23^
Zn1	0.0278 (7)	0.0212 (6)	0.0247 (6)	0.0057 (5)	0.0079 (5)	0.0030 (5)
P1	0.0177 (8)	0.0145 (8)	0.0173 (8)	0.0054 (6)	0.0056 (7)	0.0043 (6)
P2	0.0194 (8)	0.0157 (8)	0.0150 (8)	0.0029 (7)	0.0048 (7)	0.0008 (6)
O11	0.020 (2)	0.024 (2)	0.014 (2)	0.0010 (19)	0.0059 (18)	0.0015 (17)
O12	0.026 (2)	0.020 (2)	0.035 (3)	0.0118 (19)	0.016 (2)	0.015 (2)
O13	0.022 (2)	0.019 (2)	0.025 (2)	0.0082 (18)	0.0116 (19)	0.0051 (18)
O21	0.029 (3)	0.021 (2)	0.017 (2)	0.000 (2)	0.006 (2)	0.0027 (18)
O22	0.028 (2)	0.020 (2)	0.024 (2)	0.0100 (19)	0.009 (2)	0.0019 (19)
O23	0.035 (3)	0.021 (2)	0.015 (2)	0.005 (2)	0.007 (2)	0.0037 (18)
O1	0.021 (2)	0.018 (2)	0.030 (2)	0.0008 (19)	0.007 (2)	0.0003 (19)
N1	0.023 (3)	0.022 (3)	0.021 (3)	0.010 (2)	0.006 (2)	0.008 (2)
N2	0.052 (4)	0.039 (4)	0.032 (3)	0.018 (3)	0.020 (3)	0.025 (3)
C1	0.023 (3)	0.017 (3)	0.017 (3)	0.010 (3)	0.007 (3)	0.007 (2)
C2	0.029 (4)	0.023 (3)	0.019 (3)	0.010 (3)	0.002 (3)	0.004 (3)
C3	0.027 (4)	0.031 (4)	0.044 (4)	0.013 (3)	0.016 (3)	0.022 (3)
C5	0.027 (4)	0.026 (4)	0.037 (4)	0.008 (3)	0.007 (3)	0.012 (3)
C4	0.048 (5)	0.042 (5)	0.025 (4)	0.020 (4)	0.000 (3)	0.013 (3)
O1W	0.018 (2)	0.022 (2)	0.059 (3)	0.007 (2)	0.013 (2)	0.011 (2)

### Geometric parameters (Å, °)

**Table:**

Zn1—O11^i^	2.042 (4)	O1—H1	0.8201
Zn1—O11	2.042 (4)	N1—C3	1.317 (8)
Zn1—O21	2.079 (4)	N1—C5	1.379 (8)
Zn1—O21^i^	2.079 (4)	N1—C2	1.478 (7)
Zn1—O1W^i^	2.096 (4)	N2—C3	1.323 (9)
Zn1—O1W	2.096 (4)	N2—C4	1.341 (9)
P1—O11	1.501 (4)	N2—H2	0.8600
P1—O13	1.509 (4)	C1—C2	1.543 (8)
P1—O12	1.567 (4)	C2—H2A	0.9700
P1—C1	1.847 (6)	C2—H2B	0.9700
P2—O21	1.498 (4)	C3—H3	0.9300
P2—O23	1.502 (4)	C5—C4	1.352 (9)
P2—O22	1.578 (4)	C5—H5	0.9300
P2—C1	1.850 (6)	C4—H4	0.9300
O12—H12	0.8200	O1W—H1WA	0.8200
O22—H22	0.8200	O1W—H1WB	0.8200
O1—C1	1.448 (7)		
			
O11^i^—Zn1—O11	180.0	C1—O1—H1	109.4
O11^i^—Zn1—O21	89.35 (16)	C3—N1—C5	108.8 (6)
O11—Zn1—O21	90.65 (16)	C3—N1—C2	126.2 (6)
O11^i^—Zn1—O21^i^	90.65 (16)	C5—N1—C2	125.0 (5)
O11—Zn1—O21^i^	89.35 (16)	C3—N2—C4	109.7 (6)
O21—Zn1—O21^i^	180.0	C3—N2—H2	125.1
O11^i^—Zn1—O1W^i^	86.18 (18)	C4—N2—H2	125.1
O11—Zn1—O1W^i^	93.82 (18)	O1—C1—C2	106.5 (5)
O21—Zn1—O1W^i^	87.37 (18)	O1—C1—P1	108.5 (4)
O21^i^—Zn1—O1W^i^	92.63 (18)	C2—C1—P1	105.0 (4)
O11^i^—Zn1—O1W	93.82 (18)	O1—C1—P2	110.8 (4)
O11—Zn1—O1W	86.18 (18)	C2—C1—P2	112.6 (4)
O21—Zn1—O1W	92.63 (18)	P1—C1—P2	113.1 (3)
O21^i^—Zn1—O1W	87.37 (18)	N1—C2—C1	114.5 (5)
O1W^i^—Zn1—O1W	180.000 (1)	N1—C2—H2A	108.6
O11—P1—O13	115.5 (2)	C1—C2—H2A	108.6
O11—P1—O12	108.9 (2)	N1—C2—H2B	108.6
O13—P1—O12	109.9 (2)	C1—C2—H2B	108.6
O11—P1—C1	108.2 (3)	H2A—C2—H2B	107.6
O13—P1—C1	107.9 (3)	N1—C3—N2	108.0 (6)
O12—P1—C1	106.0 (3)	N1—C3—H3	126.0
O21—P2—O23	115.7 (2)	N2—C3—H3	126.0
O21—P2—O22	108.0 (3)	C4—C5—N1	106.1 (6)
O23—P2—O22	110.9 (2)	C4—C5—H5	127.0
O21—P2—C1	107.4 (3)	N1—C5—H5	127.0
O23—P2—C1	109.4 (3)	N2—C4—C5	107.4 (6)
O22—P2—C1	104.9 (3)	N2—C4—H4	126.3
P1—O11—Zn1	134.2 (2)	C5—C4—H4	126.3
P1—O12—H12	117.6	Zn1—O1W—H1WA	118.1
P2—O21—Zn1	132.6 (3)	Zn1—O1W—H1WB	130.4
P2—O22—H22	116.2	H1WA—O1W—H1WB	110.8
			
O13—P1—O11—Zn1	167.0 (3)	O12—P1—C1—P2	63.4 (3)
O12—P1—O11—Zn1	−68.8 (4)	O21—P2—C1—O1	177.3 (4)
C1—P1—O11—Zn1	46.0 (4)	O23—P2—C1—O1	−56.4 (4)
O21—Zn1—O11—P1	−30.0 (4)	O22—P2—C1—O1	62.6 (4)
O21^i^—Zn1—O11—P1	150.0 (4)	O21—P2—C1—C2	−63.6 (5)
O1W^i^—Zn1—O11—P1	−117.4 (4)	O23—P2—C1—C2	62.7 (5)
O1W—Zn1—O11—P1	62.6 (4)	O22—P2—C1—C2	−178.3 (4)
O23—P2—O21—Zn1	−172.3 (3)	O21—P2—C1—P1	55.2 (4)
O22—P2—O21—Zn1	62.8 (4)	O23—P2—C1—P1	−178.5 (3)
C1—P2—O21—Zn1	−49.8 (4)	O22—P2—C1—P1	−59.5 (3)
O11^i^—Zn1—O21—P2	−147.8 (4)	C3—N1—C2—C1	104.1 (7)
O11—Zn1—O21—P2	32.2 (4)	C5—N1—C2—C1	−72.1 (8)
O1W^i^—Zn1—O21—P2	126.0 (4)	O1—C1—C2—N1	62.2 (6)
O1W—Zn1—O21—P2	−54.0 (4)	P1—C1—C2—N1	177.1 (4)
O11—P1—C1—O1	−176.6 (3)	P2—C1—C2—N1	−59.4 (6)
O13—P1—C1—O1	57.8 (4)	C5—N1—C3—N2	0.4 (8)
O12—P1—C1—O1	−59.9 (4)	C2—N1—C3—N2	−176.3 (6)
O11—P1—C1—C2	69.9 (4)	C4—N2—C3—N1	0.3 (8)
O13—P1—C1—C2	−55.8 (4)	C3—N1—C5—C4	−0.9 (8)
O12—P1—C1—C2	−173.4 (4)	C2—N1—C5—C4	175.8 (6)
O11—P1—C1—P2	−53.3 (4)	C3—N2—C4—C5	−0.9 (9)
O13—P1—C1—P2	−178.9 (3)	N1—C5—C4—N2	1.1 (8)

Symmetry codes: (i) −*x*+1, −*y*+1, −*z*+1.

### Hydrogen-bond geometry (Å, °)

**Table:**

*D*—H···*A*	*D*—H	H···*A*	*D*···*A*	*D*—H···*A*
O22—H22···O23^ii^	0.82	1.90	2.676 (6)	159
O12—H12···O13^iii^	0.82	1.79	2.607 (6)	176
O1—H1···O23^ii^	0.82	2.28	2.910 (6)	134
O1W—H1WA···O12	0.82	2.43	3.078 (6)	137
O1W—H1WB···O13^iv^	0.82	1.94	2.745 (6)	167
N2—H2···O21^v^	0.86	1.90	2.740 (7)	164

Symmetry codes: (ii) −*x*+2, −*y*+2, −*z*+2; (iii) −*x*+2, −*y*+2, −*z*+1; (iv) *x*−1, *y*, *z*; (v) −*x*+2, −*y*+1, −*z*+2.
